# Western Iowa Veterinary Medical Association

**Published:** 1892-03

**Authors:** L. U. Shipley

**Affiliations:** Secretary


Meeting then adjourned.

AUSTIN PETERS, *Secretary.*

---

*Western Iowa Veterinary Medical Association.*—The fourth meeting of the Western Iowa Veterinary Medical Association, was held at Carroll, January 20th, 1892. The President, S. H. Johnson, in the chair; the following members were present: S. H. Johnston, G. A. Jonson and G. C. Williams, with Dr. L. U. Shipley as a visitor. The minutes of the previous meeting was read by the Secretary, and approved. Among the communications were letters of regrets from several members, also an invitation from Dr. W. C. McClanahan to the members of the Association to forward to the Veterinary Department of the Iowa Agricultural College, any or all pathological specimens which they desired to have investigated; the invitation was accepted and placed on file.

The delegate to the Iowa State Veterinary Medical Association, reported that he had secured the adoption by that Association of a bill for legislation in accordance with the wishes of the members of this Association, as expressed at the meeting held on October 21st, also the adoption of a service fee bill. Dr. L. U. Shipley was admitted to membership, after which a general discussion was indulged in. Among other things, Dr. S. H. Johnston recommended the use of acetanilide in tympanites. Dr. Shipley presented a report of cases, namely: Pericarditis, posterior displacement of the caecum, and prolapsus of the rectum, which were fully discussed. Dr. S. H. Johnston read a paper on Odontomes of Cattle, and advanced the idea that many so-called cases of lumpy jaw were in reality nothing but odontomes.

The election of officers resulted as follows: President, G. J. Gibson; Vice-president, G. C. Williams; Secretary and Treasurer, L. U. Shipley. The meeting then adjourned, to meet at Carroll, at the call of the Secretary.

L. U. Shipley, *Secretary.*

